# Rectal ectopic pregnancy: A case report

**DOI:** 10.1016/j.ijscr.2024.109798

**Published:** 2024-05-23

**Authors:** Muhammed Saif, Shaymaa Alahmar, Ahmed Saif, Nahed Al Halabi, Dema Adwan, Imad Altanoukhi

**Affiliations:** a Faculty of Medicine, Al-Sham Private University, Damascus, Syria; b Faculty of Medicine, Damascus University, Damascus, Syria; c Department of Obstetrics and Gynecology, Maternity University Hospital, Damascus, Syria; d Emergency Department, Maternity University Hospital, Damascus, Syria; e Department of Obstetrics and Gynecology, Al-Sham Private University, Damascus, Syria

**Keywords:** Rectal pregnancy, Abdominal pregnancy, Ectopic pregnancy, Abdominal pain, Appendicitis, Case report

## Abstract

**Introduction:**

Abdominal pregnancy is an extremely rare form of ectopic gestation, and it presents with pelvic pain, severe bleeding, or remain asymptomatic. Its Risk factors include previous ectopic pregnancies, cesarean section, smoking, pelvic inflammatory disease, using intrauterine devices (IUD), and assisted reproductive techniques (ARTs). Accurate diagnosis of rectal ectopic pregnancy remains challenging due to the lack of well-established diagnostic criteria.

**Case presentation:**

A 25-year-old woman presented to the emergency department with a 2-day history of unresponsive lower abdominal pain and nausea. Ultrasound imaging revealed a normal-sized uterus with endometrial thickness, fluids, and clots in the abdominal cavity, but no intrauterine gestational sac was detected. Based on the clinical presentation, ectopic pregnancy was suspected. During laparotomy, the placenta and fetal tissue remnants were found on the anterior wall of the upper third of the rectum.

**Discussion:**

Abdominal ectopic pregnancy is a high-risk condition that can manifest with gastrointestinal symptoms such as nausea, vomiting, constipation, as well as abdominal and pelvic pain. These variable symptoms underscore the importance of considering rectal ectopic pregnancy as a differential diagnosis and ruling it out to prevent life-threatening complications, including severe bleeding.

**Conclusion:**

Due to its rarity, diverse presentation, and similarity to other conditions, diagnosing rectal ectopic pregnancy and determining the appropriate management can be challenging. Physicians should be aware of this specific type of ectopic pregnancy to enable early-stage diagnosis and provide optimal care.

## Highlights

• Rare case of rectal ectopic pregnancy
• Diagnosing rectal ectopic pregnancy remains challenging due to the lack of well-established diagnostic criteria.
• Awareness and suspicion are crucial for the diagnosis of rectal ectopic pregnancy.
• Early-stage diagnosis and optimal care are essential for managing rectal ectopic pregnancy effectively.

## 1 Introduction

Ectopic pregnancy is a condition where the implantation of a developing blastocyst occurs outside the endometrial cavity of the uterus, accounting for 2 % to 3 % of normal pregnancies. Despite advancements in diagnosis and management, ectopic pregnancies still contribute by 4 % to 10 % of total pregnancy-related deaths and often result in a higher risk of ectopic site gestations in subsequent pregnancies [1,2]. Several risk factors have been identified, including previous ectopic pregnancies, cesarean section, smoking, pelvic inflammatory disease, using intrauterine devices (IUD), and assisted reproductive techniques (ARTs) [3]. Symptoms of ectopic pregnancy can range from asymptomatic to pelvic pain or hemorrhagic shock [4]. Abdominal pregnancy is an extremely rare form of ectopic gestation, occurring in approximately 0.9 % to 1.4 % of all ectopic pregnancies [5]. While transvaginal ultrasonography and serum β-hCG measurement are commonly used for diagnosis, additional methods such as abdominal X-ray, computed tomography, magnetic resonance imaging, and diagnostic laparoscopy may be necessary. Although some medical treatments have been reported, surgery remains the primary treatment for most cases of abdominal pregnancy [6]. This report presents a rare case of abdominal ectopic pregnancy attached to the anterior wall of the rectum. Despite multiple diagnostic methods, a definitive diagnosis was challenging to establish, emphasizing the importance of establishing clear guidelines and standards for managing patients with Rectal ectopic pregnancy. This work has been reported in line with the SCARE criteria [7].

## 2 Case presentation

A 25-year-old woman, gravida 1, para 1 (vaginal birth), presented to the emergency department with a 2-day history of unresponsive lower abdominal pain and nausea. She had no surgical or allergic history and she doesn't use any contraceptive methods. Her exact last menstrual date was unknown due to breastfeeding. The patient was conscious and vitally stable.

Abdominal and pelvic ultrasound revealed a normal-sized uterus with endometrial thickness of 15 mm, an empty uterus, and a small amount of free fluid and blood clots in the pouch of Douglas. Additionally, a simple cyst measuring 20 × 30 mm was observed in the right uterine adnexa which coincides with a corpus luteum cyst. However, we noticed a heterogenous echogenic mass measuring 40 × 35 mm with no fetal cardiac activity, but its exact origin (fallopian tube, ovary, or elsewhere) could not be accurately determined.

The patient's blood pressure was 110/70 mmHg, pulse rate was 89 beats per minute, hemoglobin (Hb) level was 9.9 g/dl (normal range: 12.3–15.3 g/dl), and serum β-hCG level was 3458 IU/l (normal range for non-pregnant women is less than 5 IU/l). Prothrombin time (PT) was 14 s (normal range: 11 to 13,5 s), and activated partial thromboplastin time (PTT) was 25 s (normal range: 30–40 s), white blood cells count (WBC) was 7 × 10^3^ \ mm^3^ (normal range: 4–11 × 10^3^ \ mm^3^). With the suspicion of ectopic pregnancy and the patient being hemodynamically stable with normal liver and kidney function tests, a 50 mg/m^2^ single dose of intramuscular methotrexate was prescribed, and β-hCG levels were monitored every 48 h.

Two days later, the patient experienced sudden weakness, paleness, and severe abdominal pain. Vital signs indicated a blood pressure of 90/60 mmHg and a pulse rate of 120 beats per minute. Laboratory results showed a decrease in hemoglobin level to 7.9 g/dl, PTT of 32 s, and PT of 56 %. Ultrasound imaging revealed a normal-sized uterus with endometrial thickness, along with fluids and clots filling the abdominal cavity and reaching the Morison pouch.

**Fig. 1:**
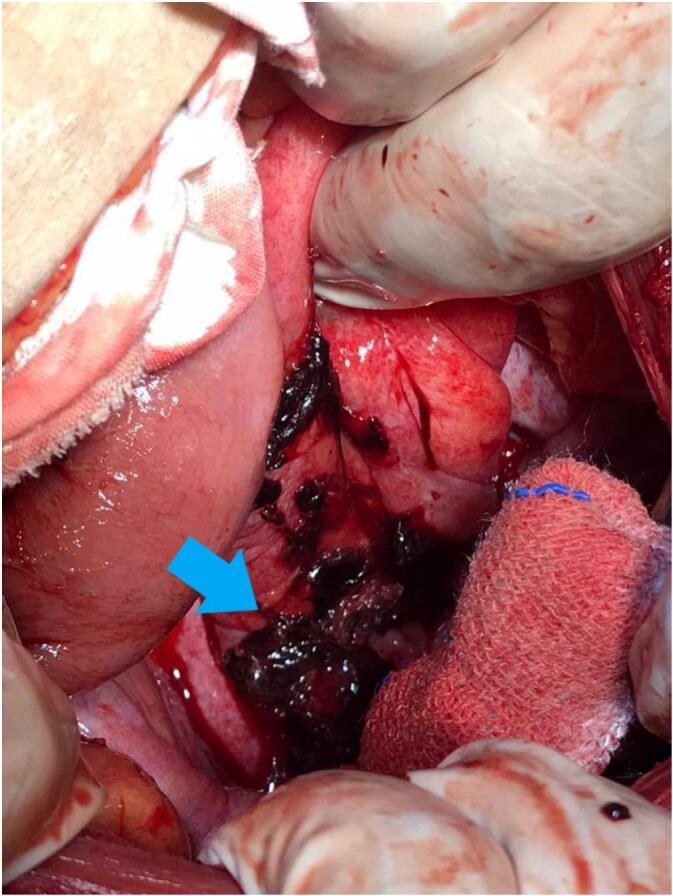
The attachment site of the placenta on the anterior wall of the rectum with blood clots (arrow).

Based on the clinical, laboratory, and imaging findings, and because the patient is vitally unstable and is close to hemorrhagic shock, the decision was made to improve the patient's general condition and proceed with immediate surgical intervention. During laparotomy, both fallopian tubes were normal, right ovarian cyst was observed, along with abundant blood and clots. A placenta and remnants of fetal tissue were identified on the anterior wall of the upper third of the rectum. Hemostasis was achieved, and surgical removal of the ectopic pregnancy was performed (Fig. 1). General surgeons examined the lower gastrointestinal tract and did not find any injuries or perforations; however, an inflamed appendix was also removed.

Histological examination results confirmed the diagnosis of rectal ectopic pregnancy. After a few days, the patient made a complete recovery and was discharged from the hospital. β-hCG values were monitored, and they decreased to 300 IU/l after one week, with subsequent measurements turning negative after three weeks.

## 3 Discussion

Abdominal ectopic pregnancy is a life-threatening condition, with a higher risk of mortality compared to other types of ectopic pregnancies [5]. With maternal mortality rates about 5 %, the severity of the condition varies depending on the location of the pregnancy, and the risk of severe bleeding is a significant concern [8].

Diagnosing rectal ectopic pregnancy can be challenging, and there is no established set of diagnostic criteria. Reported cases in the literature have presented with various symptoms, including abdominal pain, vaginal discharge, dysuria, diarrhea, rectal pressure, and menstrual cycle abnormalities [[9], [10], [11], [12]]. It is important for physicians to consider the possibility of rectal pregnancy and rule it out to avoid serious complications such as severe bleeding. It is worth noting that some cases of rectal ectopic pregnancy have occurred in women who underwent in vitro fertilization [10,11], suggesting it may be a prominent risk factor. Acute appendicitis is one of the most common acute surgical conditions. However, there is a confusion sometimes between the diagnosis and management in pregnant patients. The management of appendicitis during pregnancy is often a collaborative effort between the surgical and obstetric teams. The optimal management aims to diagnose appendicitis in pregnant patients early and provide prompt treatment to prevent any risks to the patient, especially since the physiological changes of the pregnancy pose both diagnostic and therapeutic challenges for the physician [13]. However, in our case, the presence of appendicitis was discovered during the laparotomy, highlighting the need to consider other potential conditions.

Ultrasound is commonly used for diagnosing abdominal ectopic pregnancy, but its accuracy rate can be limited, with some studies reporting rates as low as 50 % [14]. However, the diagnosis of abdominal pregnancy can still be made preoperatively using abdominal and pelvic ultrasound, but without accurately determining its exact location. The essential criteria for diagnosing abdominal pregnancy according to Allibone et al. include:

1) demonstration of a fetus in a gestational sac outside the uterus, or the depiction of an abdominal or pelvic mass identifiable as the uterus separate from the fetus.
2) failure to see a uterine wall between the fetus and urinary bladder.
3) recognition of a close approximation of the fetus to the material abdominal wall.
4) localization of the placenta outside the confines of the uterine cavity. [15]

In this case, criteria 1, 3, and 4 were confirmed, which is why the diagnosis relied on ultrasound before surgery. The availability of advanced imaging techniques such as computed tomography (CT) and magnetic resonance imaging (MRI) has improved the accuracy and safety of diagnosing abdominal pregnancy. These imaging modalities help locate the ectopic pregnancy, assess its anatomical relationships, and evaluate its vascular supply, facilitating surgical intervention and reducing complications [8].

Abdominal pregnancies can be categorized as primary or secondary. The majority are secondary, resulting from the rupture of a tubal or ovarian pregnancy, while primary abdominal pregnancies occur when fertilization takes place within the abdominal cavity. In our case, the diagnosis aligns with the criteria described by Studdiford in 1942 [16], which includes:

1. Presence of normal tubes and ovaries with no evidence of recent or past pregnancy.
2. No evidence of uteroplacental fistula.
3. The presence of a pregnancy related exclusively to the peritoneal surface and early enough to eliminate the possibility of secondary implantation after primary tubal abortion.

Abdominal pregnancies are associated with delayed symptom onset and increased morbidity and mortality due to their larger size compared to intratubal pregnancies [17]. They can present with gastrointestinal symptoms, abdominal and pelvic pain, bleeding, anemia, jaundice, oliguria, and toxic-infectious syndrome [18].

Choosing surgical treatment (laparoscopic or laparotomy) depends on the patient's condition, whereas medical therapy -such as systemic methotrexate chemotherapy or ultrasound-guided injection of potassium chloride- may be considered for early abdominal pregnancies [6]. However, the optimal management of early abdominal pregnancy has always been controversial. These cases can be treated medically, surgically, or through a combination of both. The management is determined based on the gestational age at diagnosis, fetal cardiac activity, and the clinical condition of the mother. In our case, upon admission, the patient met the criteria for initiating medical treatment. However, when a life-threatening hemorrhage occurred, along with hemodynamic instability and the proximity of the pregnancy to vital organs, the optimal approach was surgical exploration of the abdomen to control the bleeding and improve the clinical condition [19,20]. The choice between laparoscopic or open surgery is also a subject of debate and primarily depends on the patient's clinical condition.

## 4 Conclusion

Rectal ectopic pregnancy is an extremely rare condition that requires awareness and suspicion for diagnosis. In addition to traditional diagnostic methods such as ultrasound and monitoring β-hCG levels, modern imaging techniques like CT and MRI can aid in early detection and provide a better understanding of the potential of surgical intervention and complications. It is important to consider other adjacent anatomical structures that may present with symptoms similar to ectopic pregnancy.

## Consent

Written informed consent was obtained from the patient for publication of this case report and accompanying images. A copy of the written consent is available for review by the Editor-in-Chief of this journal on request.

## Ethical approval

Not required for case reports in our hospital. Single case reports are exempt from ethical approval in our institution.

## Funding

The authors declare no source of funding for this manuscript from any organization or any institution.

## Author contribution

MS, SA, AS, and NAH reviewed the literature and wrote the manuscript. DA and IA supervised and reviewed the manuscript. All authors read and approved the final manuscript.

## Guarantor

Dr. Dema Adwan and Dr. Imad altanoukhi.

## Registration of research studies

Not applicable.

## Declaration of competing interest

All of the authors declared that they have no conflict of interest.
